# Investigating Kinetic Characterization of Variable-Rate Fatigue Process of SBS Asphalt Mixture with Physical Gel Structures

**DOI:** 10.3390/gels12090839

**Published:** 2026-09-13

**Authors:** Chenze Fang, Jiahao Yang, Menghao Wang, Hongbin Zhu, Pingfan Hu

**Affiliations:** 1 School of Civil Engineering and Transportation, North China University of Water Resources and Electric Power, Zhengzhou 450045, China; fangchenze@ncwu.edu.cn (C.F.); 15639598669@163.com (J.Y.); 2 School of Civil Engineering, Suzhou University of Science and Technology, Suzhou 215009, China; 3 College of Transportation Engineering, Dalian Maritime University, Dalian 116026, China; 4 Artie McFerrin Department of Chemical Engineering, Texas A&M University, College Station, TX 77843-3122, USA; pingfanhu@tamu.edu

**Keywords:** asphalt pavement engineering, SBS asphalt mixture, variable-rate fatigue process, kinetics theory, activation energy

## Abstract

The fatigue of an SBS asphalt mixture with physical gel structures under repeated loading is a variable-rate mechanical process. However, there is a lack of a mechanical characterization mechanism to accurately quantify this process. This study aims to propose a method for mechanically characterizing the variable-rate fatigue process of the SBS asphalt mixture based on kinetics theory. First, indirect tensile monotonic and repeated-loading tests with advantages of easy specimen fabrication, good test repeatability, and widespread use in pavement fatigue evaluation were conducted at 15 °C, 20 °C, and 25 °C to obtain the mechanical response of SBS asphalt mixtures. Then, a permanent strain model accounting for damage of the SBS asphalt mixture was established based on viscoelastic damage theory. Finally, a fatigue damage kinetic model was developed. The parameters, represented in terms of lumped damage sensitivity exponent (*β*) and fatigue activation energy (*E*_a_), were determined to quantitatively characterize the variable-rate fatigue behavior of the SBS asphalt mixture. The results show that the established permanent strain model accounting for damage can accurately capture the nonlinear evolution of fatigue damage and permanent strain in SBS asphalt mixtures. The parameter *β* can serve as a reliable mechanical indicator for quantifying the fatigue process rate. The fitted kinetic parameter *E*_a_ can reasonably characterize the magnitude of the energy barrier governing the temperature-dependent variable-rate fatigue process. Macroscopically, the SBS asphalt mixture presents an elevated fatigue damage energy threshold compared with the base mixture. According to existing literature, this difference may be associated with three-dimensional physical gel structures from the SBS polymer. Within the scope of the present test conditions, kinetics theory shows potential as a theoretical framework for quantifying and characterizing the variable-rate fatigue behavior observed for SBS asphalt mixtures.

## 1. Introduction

Although the elastomeric SBS polymer effectively enhances the fatigue resistance of asphalt mixtures, fatigue cracking can still occur in SBS asphalt pavements under long-term vehicular cyclic loading and shorten pavement service life [1,2,3].

As one prevailing modifier material, SBS has gained extensive application in high-grade pavement engineering [4,5,6]. It can form three-dimensional physical gel networks within the asphalt matrix through microphase separation, endowing the mixture with excellent elastic recovery and resistance to fatigue cracking. Under the sustained action of long-term alternating loadings, fatigue damage within the SBS asphalt mixture exhibits a nonlinear variable-rate evolution. Accurately quantifying such variable-rate fatigue process remains a critical issue in the field of pavement materials that urgently needs to be addressed, and this work is vital for refining the material formulation of SBS asphalt mixtures and for lengthening the service lifespan of SBS asphalt pavements.

Most existing studies characterize the fatigue properties of asphalt mixtures using three approaches: phenomenological methods, energy-based methods, and continuous damage mechanics [7,8,9,10]. Phenomenological methods rely on a power-law relationship between load amplitude and fatigue life to perform macroscopic fitting. Nevertheless, these methods can only yield empirical formulas for fatigue life and fail to reflect the intrinsic kinetic behavior of fatigue damage [11,12,13]. Traditional energy methods use total dissipated energy as an evaluation metric, making it difficult to isolate the purely viscoelastic reversible dissipated energy that does not contribute to damage and accurately distinguish the dissipated energy directly related to fatigue cracking [14,15,16]. Fracture mechanics can describe the behavior of macroscopic crack propagation, but it struggles to capture the evolutionary characteristics during the early stages of damage initiation, resulting in significant limitations in its applicability [17,18,19,20]. Viscoelastic continuous damage mechanics provides a unified description of the entire process of crack initiation and propagation. The damage evolution equation based on energy dissipation can establish a fundamental theoretical framework for the quantitative analysis of nonlinear damage in asphalt materials. However, such a framework does not incorporate a kinetic quantification approach and lacks methods for quantitatively characterizing the fatigue development rate and energy threshold for fatigue processes.

As a typical sol–gel viscoelastic medium, asphalt combines the flow characteristics of a sol with the constraining effect of a cross-linked gel networks [21,22]. The key feature that distinguishes the SBS asphalt from base asphalt is its three-dimensional physical polymer gel networks. Such structure alters the energy thresholds for the initiation and propagation of internal damage, ultimately resulting in significant differences in the fatigue damage evolution rates and temperature sensitivity between the two types of mixture. Under equivalent temperature and load conditions, SBS asphalt mixtures exhibit a more gradual progression of fatigue damage and a significantly longer fatigue life. Most existing fatigue-related mechanical models focus on fitting mechanical responses, but they do not sufficiently explore the kinetic nature of variable-rate fatigue damage in the SBS asphalt mixture, making it difficult to establish a quantitative relationship between the fatigue process rate, damage energy thresholds, and the fatigue performance of the SBS asphalt mixture.

Kinetic theory employs rate parameters and activation energy as two core metrics to quantitatively characterize the rate and energy barriers of physical reactions. This theory has now been progressively extended to mechanistic analyses of aging, self-healing, and plastic deformation in asphalt binders. Li et al. [23] developed a kinetics-based model of fatigue crack growth rate in bituminous material. Fang et al. [24] extended this kinetic framework to solid-waste-modified asphalt, demonstrating that waste-battery-powder modifiers enhance fatigue damage activation energy and induce toughening performance. Fang et al. [25] further adopted this kinetic concept for plant-fiber-modified asphalt, and illustrated the correlation between micro-grooved fiber morphology and elevated fatigue damage energy thresholds. Previous studies have established a kinetic characterization framework for SBS binders, confirming that three-dimensional physical polymer gel networks can significantly increase the energy threshold at which fatigue damage occurs in the binder [26]. However, such research has been limited to the scale of asphalt binders, and has not been extended to asphalt mixture systems that include an aggregate skeleton. There is a lack of quantitative kinetic methods of the SBS asphalt mixture established through indirect tensile repeated loading tests in the laboratory, making it impossible to provide a complete quantitative explanation of the relationship between macroscopic fatigue mechanical responses and the energy thresholds of variable-rate fatigue processes in the SBS asphalt mixture.

According to extensive existing literature, SBS polymer can form reversible three-dimensional physical gel networks within asphalt binder via micro-phase separation [6,14,21,22]. Such micro-structures are reported to modify the viscoelastic response, raise damage resistance, and alter temperature-dependent mechanical behaviors of asphalt-based materials. Therefore, the physical gel network mechanism provides a potential interpretive framework for understanding the difference in fatigue evolution between SBS and base asphalt mixtures. It should be emphasized that the current study focuses on macroscopic mechanical and kinetic characterization of mixture scale fatigue behavior, and direct experimental observation of gel network micromorphology is not included in our test program. Interpretations related to gel network structures in subsequent sections are derived from previous publications instead of direct experimental evidence obtained in this investigation.

Given these concerns, this study focuses on SBS asphalt mixture and conducts indirect tensile monotonic and repeated loading tests at 15 °C, 20 °C, and 25 °C to collect comprehensive fatigue mechanical response data under cyclic loading. By integrating viscoelastic damage theory with kinetics theory, a mechanical characterization framework tailored to the variable-rate fatigue behavior of the SBS asphalt mixture has been established. The lumped damage sensitivity exponent and fatigue activation energy were employed to quantify the rate and energy barriers of fatigue processes in the SBS asphalt mixture. A kinetics-based fatigue quantification framework of SBS asphalt mixtures was established. Macro-scale fatigue observations are further discussed with reference to previously reported physical SBS polymer network mechanisms. This can provide a theoretical basis for material optimization and fatigue life prediction of asphalt pavements.

## 2. Results and Discussion

### 2.1. Permanent Strain Response and Variable-Rate Fatigue Damage Evolution of SBS Asphalt Mixtures Under Repeated Loading

#### 2.1.1. Permanent Strain Response of SBS Asphalt Mixture

**Figure 1:**
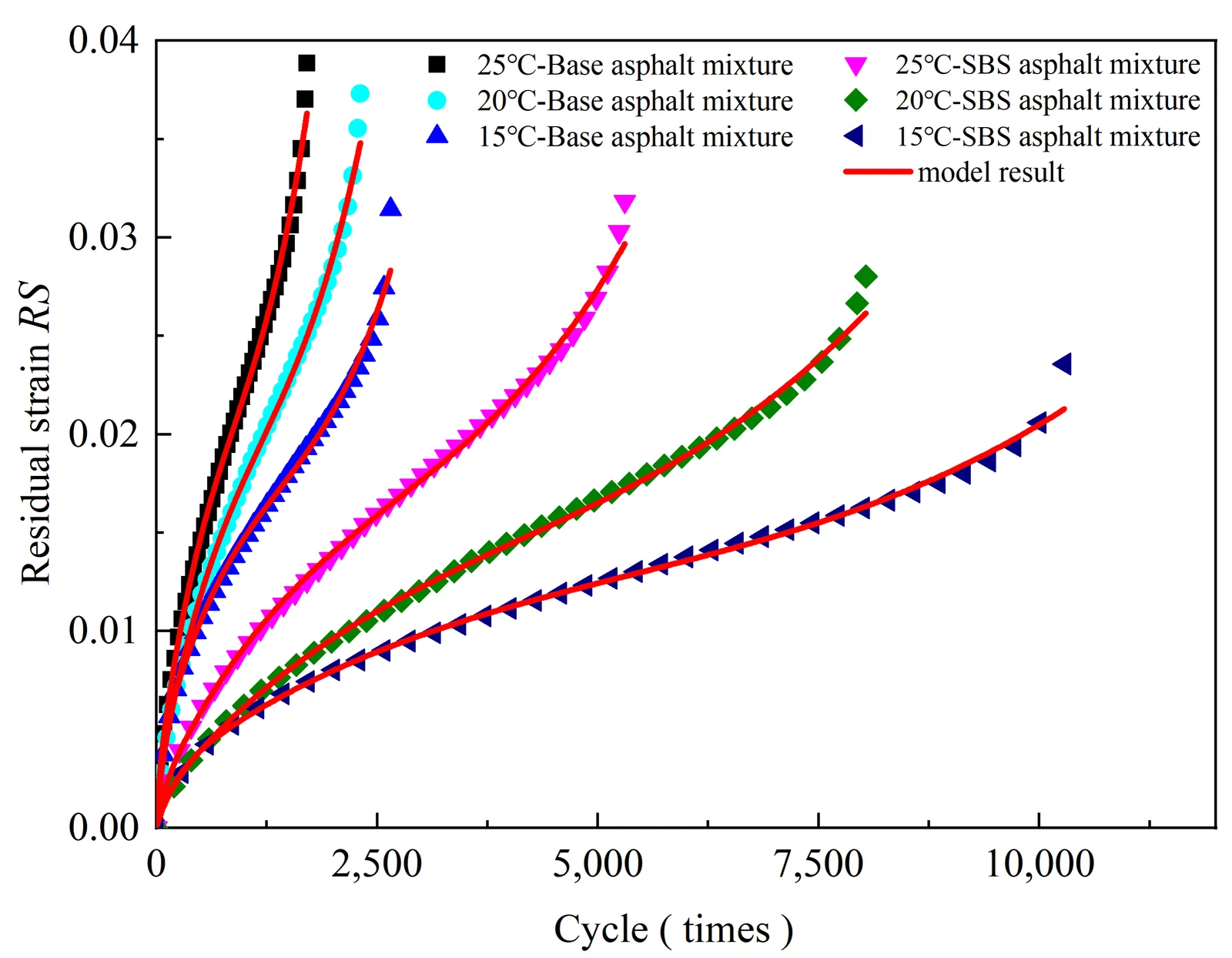
Matching permanent strain results model for SBS and base asphalt mixtures.

The permanent strain results of SBS and base asphalt mixtures at different temperatures are shown in Figure 1. In this paper, a permanent strain model that accounts for damage of the SBS asphalt mixture was established based on viscoelastic damage theory. The corresponding detailed derivation is presented in Section 4.4. The permanent strain curves under different working conditions were matched using the established permanent strain model, and Figure 1 and Table 1 shows the matching results. As shown in Figure 1 and Table 1, the established permanent strain model accounting for damage is capable of capturing the nonlinear development of permanent strain for the SBS asphalt mixture throughout the damage progression.

**Figure 2:**
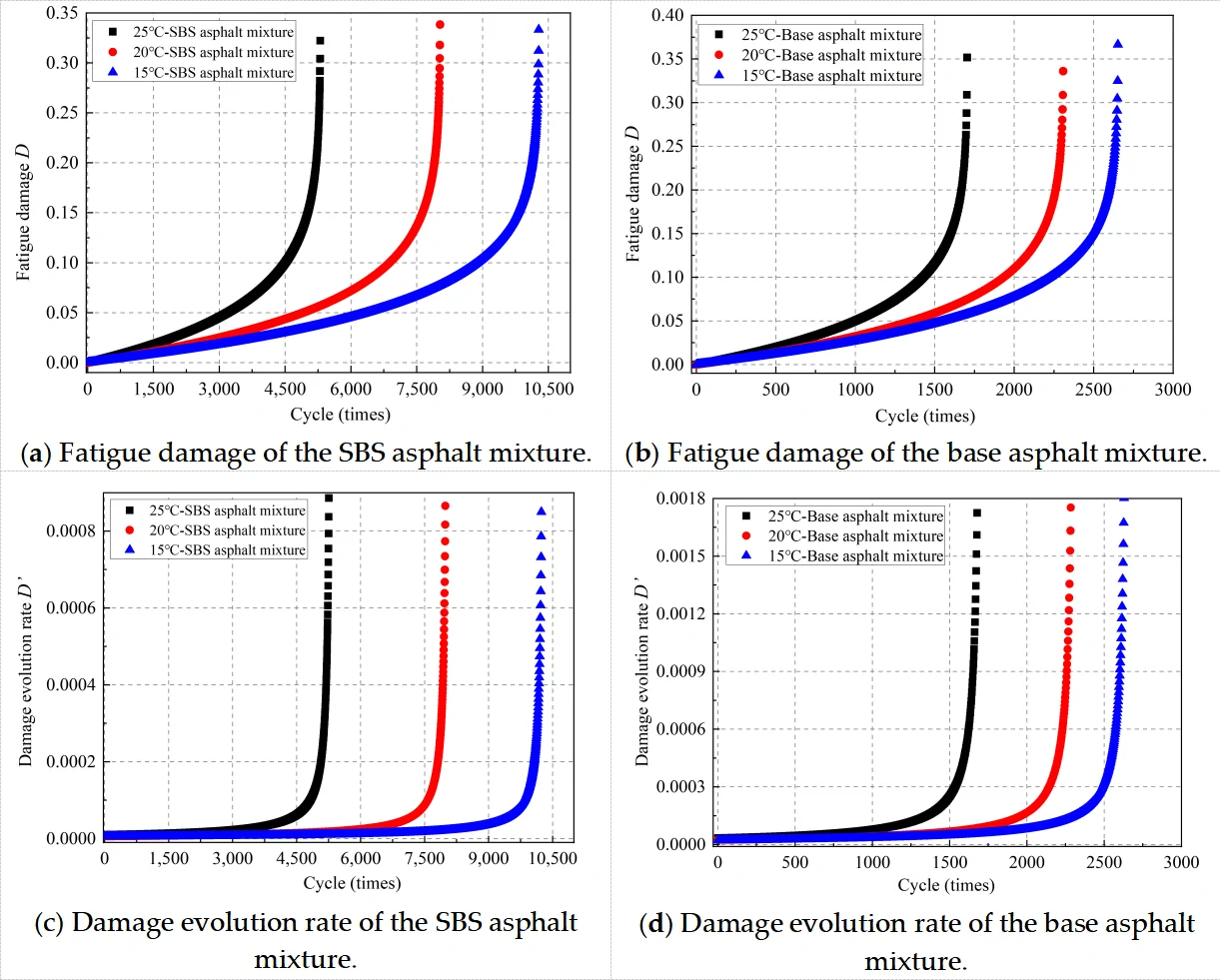
Fatigue damage and damage evolution rate of the SBS and base asphalt mixtures under repeated tensile loading.

As illustrated in Figure 2 and Table 1, the nonlinear development of permanent strain for SBS asphalt mixtures proceeds through three distinct phases. When temperature rises from 15 °C to 25 °C, the permanent strain rate presents an obvious upward tendency. At lower temperatures, asphalt mixtures are capable of sustaining a larger number of load cycles prior to the onset of viscous flow, which yields a reduced permanent strain rate. With elevated temperature, viscous flow deformation induced per load cycle grows, which leads to a positive correlation between permanent strain rate and temperature [27,28,29]. Furthermore, SBS asphalt mixtures possess a smaller permanent strain rate compared with base asphalt mixtures. According to previous literature, this behavior is inferred to be partly related to three-dimensional physical gel networks formed by SBS modifiers within the asphalt mixture matrix.

#### 2.1.2. Variable-Rate Fatigue Damage Evolution of SBS Asphalt Mixture

Previous studies have demonstrated that damage evolution formulations defined by dissipated energy offer reliable characterization for fatigue damage progression in viscoelastic materials [30,31,32], as shown below:

(1) $$\frac{dD}{dN}={\left(\frac{\partial W_{N}}{\partial N}\right)}^{\beta }$$

where *D* is the damage variable, which is defined as the ratio of defect area to the total cross-sectional area of the specimen.

Carrying out integration on the preceding expression yields the mathematical form of the viscoelastic fatigue damage model shown below:

(2) $$D=1-{\left(1-\frac{N}{N_{f}}\right)}^{\frac{1}{1+2\beta }}$$

where *N_f_* is the fatigue life.

By substituting the calibrated damage model parameters into the fatigue damage model shown in Equation (2), the damage curves of the SBS asphalt mixture under repeated loading were obtained. Furthermore, the slope of these damage curves yields the damage evolution rate. The resulting damage and damage evolution rate curves under various testing conditions are presented in Figure 2.

As illustrated in Figure 2, the fatigue damage development of the SBS asphalt mixture follows a nonlinear tendency, with its rate grows continuously. When the test temperature increases from 15 °C to 25 °C, greater cumulative fatigue damage is generated under identical loading cycles, which demonstrates that the damage growth rate is positively related to temperature. This phenomenon stems from the material’s viscoelastic characteristics varying with temperature [33,34]. Under elevated-temperature conditions, the asphalt mixture produces an increased phase angle, signifying dominant viscous mechanical performance and accelerating the propagation of fatigue cracks. In contrast, at lower temperatures, the elastic response becomes more dominant, leading to a slower fatigue cracking rate; consequently, the fatigue damage accumulated per loading cycle and the corresponding damage evolution rate are both reduced. Furthermore, compared with the base asphalt mixture, the SBS mixture exhibits a consistently lower fatigue damage evolution rate.

From the above analysis, the fatigue behavior of the SBS asphalt mixture subjected to cyclic loading can be described as an intricate physical damage process with a variable rate. Compared with the base asphalt mixture, the SBS mixture exhibits superior anti-cracking performance, delayed fatigue damage accumulation per loading cycle, a reduced phase angle, and a more dominant elastic response, leading to a slower fatigue damage evolution rate. These macroscopic differences may be partly interpreted by the physical gel network structures formed by SBS modifiers within the asphalt mixture.

### 2.2. Kinetic Characterization of Variable-Rate Fatigue Process of SBS Asphalt Mixture

#### 2.2.1. Kinetics Theory

For any physical or chemical process within a given system, two fundamental kinetic parameters govern its behavior: the rate at which the process proceeds and the energetic difficulty with which it occurs. A thorough understanding of these two parameters is essential for accurately characterizing the process [23,24]. Kinetics theory provides a mathematical description of how system properties evolve over time. In other words, kinetic models are developed to precisely depict physical or chemical reaction processes, and to enable a scientifically rigorous characterization and evaluation of these processes by quantifying both their rates and their inherent energetic barriers. In the field of road engineering, the Arrhenius kinetic model shown in Equation (3) is widely adopted for its ability to accurately describe the temperature-dependent evolution of representative physical process rates [25,26].

(3) $$k=Ae^{\frac{-E_{a}}{RT}}$$

where *k* is the rate constant; *E*_a_ is the activation energy (J·mol^−1^), *T* is the absolute temperature (*k*), *R* is the universal gas constant (J·mol^−1^), and A is the pre-exponential factor.

In kinetics theory, the representative rate (*k*) is typically employed to quantify the speed at which a physical or chemical process proceeds from its initial state to equilibrium. By correlating temperature with the representative rate through kinetics equations, the minimum energy required for the process to occur, i.e., the activation energy (*E*_a_), can be determined. Theoretically, the activation energy of a given physical or chemical process reflects the intrinsic properties of the system and depends solely on the material characteristics involved. Consequently, activation energy serves as a measure of the energetic difficulty with which the process occurs: a higher activation energy corresponds to a greater energy threshold, indicating that the process is less likely to take place.

The Arrhenius-type treatment adopted in this study is grounded in the kinetics principle that the temperature dependence of a rate-related parameter can be described by an exponential relationship with the inverse of absolute temperature. Within this framework, activation energy serves as a metric for quantifying the energy barrier of the rate-governed process. In the present study, this formalism is extended to fatigue damage evolution by treating the lumped index *β* as a lumped damage sensitivity exponent. The resulting *E*_a_ thus reflects the relative energy barrier magnitude under the specific test conditions, providing a quantitative basis for comparing the temperature susceptibility of different asphalt mixtures within the kinetics framework.

It should be noted that the classical Arrhenius equation, Equation (3), describes forward-reaction-type rate constant *k*, which decreases with decreasing temperature. The lumped damage sensitivity exponent *β* in this study exhibits opposite temperature-dependent behavior and cannot be directly substituted into Equation (3). A tailored Arrhenius-type formulation is therefore adopted for *β*, as elaborated in Section 2.2.3.

#### 2.2.2. Quantitative Analysis of Fatigue Process Rate of SBS Asphalt Mixture

The fatigue damage process of the SBS asphalt mixture under repeated loading is a complex physical process with a variable rate. To quantify the rate at which this process proceeds, the damage evolution equation characterized by dissipated energy shown in Equation (4) is employed, yielding the following:

(4) $$\ln(\frac{dD}{dN})=\beta \ln(\frac{\partial W_{N}}{\partial N})$$

Equation (4) reveals a linear relationship between $\ln(\frac{dD}{dN})$ and $\ln(\frac{\partial W_{N}}{\partial N})$, the slope of which is equal to *β*. This parameter *β* effectively reflects the damage evolution rate of the material under specific testing conditions.

As the fatigue life fraction increases from 0 to 1, the damage progressively evolves toward the failure threshold, during which the damage evolution described by the damage model shown in Equation (2) is governed directly by *β*. This finding indicates that *β* can serve as the lumped damage sensitivity exponent for asphalt under cyclic loading, quantitatively characterizing the rate at which the fatigue damage process proceeds.

The *β* of the SBS asphalt mixture under various testing conditions is presented in Table 2. As the temperature decreases from 25 °C to 20 °C and further to 15 °C, *β* increases from 8.45255 to 8.70471 and 8.90380, respectively—a total increase of 5.339%. By comparison, the *β* value for the base asphalt mixture increases by only 0.347% over the same temperature range. These findings demonstrate that reducing the test temperature leads to a consistent increase in the *β*.

Equation (4) gives the linear relation between the dissipated-energy-related term and the damage variable, and its slope equals *β*. Adopting lumped model-fitted parameters as representative indicators for rate-dependent physical processes represents a well-established peer-reviewed paradigm within asphalt material kinetics research. This paradigm has been validated in previous investigations concerning aging, recovery, and healing behavior for various asphalt mixture types [23,24]. Following this established research paradigm, *β* is defined as a lumped damage sensitivity exponent derived from damage-evolution-model fitting, rather than a direct macroscopic real damage rate constant.

Under given loading conditions, the magnitude of *β* couples multiple internal visco-damage characteristics of the asphalt mixture. An apparent counter-intuitive phenomenon is observed: as temperature decreases, the numerical value of *β* increases, while the macroscopic actual fatigue damage evolution slows down and the fatigue life gets longer. This is because the real fatigue damage rate is comprehensively governed by *β* and other viscoelastic damage state variables, rather than being solely proportional to *β*. Hence, *β* can still serve as a lumped damage sensitivity exponent to describe the temperature-dependent feature of the variable-rate fatigue process of asphalt mixtures [24,25,26]. Further fully quantitative decoupling and correlation between *β* and the real damage rate will be investigated in future research.

The macroscopic fatigue-damage evolution rate $\frac{dD}{dN}$ is governed jointly by both *β* and the per-cycle dissipated energy increment $\frac{\partial W_{N}}{\partial N}$. Under low temperature circumstances, *β* becomes larger (higher damage sensitivity to energy input), meanwhile material stiffness rises and per-cycle dissipated energy input $\frac{\partial W_{N}}{\partial N}$ decreases substantially. The suppressing effect from reduced dissipated energy dominates, leading to slower macroscopic damage progression and longer fatigue life, even though *β* itself increases numerically. *β* cannot be used alone to judge the real magnitude of damage-evolution speed.

#### 2.2.3. Quantitative Analysis of Energy Threshold of Fatigue Process of SBS Asphalt Mixture Based on Fatigue Activation Energy

For the damage model shown in Equation (2), *β* is adopted as the lumped damage sensitivity exponent for characterizing the damage evolution of the SBS asphalt mixture. By combining Equations (2) and (3),the kinetic model for damage evolution in SBS asphalt mixture is derived as follows:

(5) $$D=1-{\left(1-\frac{N}{N_{f}}\right)}^{\frac{1}{1+2Ae^{\frac{-E_{a}}{RT}}}}$$

where *E*_a_ is the fatigue activation energy (J·mol^−1^).

Fatigue activation energy *E*_a_ is a kinetic fitting parameter obtained from the temperature dependence of *β.* It characterizes the magnitude of the energy barrier for temperature-dependent physical fatigue damage evolution of an asphalt mixture, rather than a directly measured physical minimum-energy threshold for damage initiation. Larger *E*_a_ values correspond to higher energy barriers that need to be overcome for fatigue damage development under varying-temperature conditions [24,25,26]. It should be noted that the present study only delivers macroscopic mechanical and kinetic test results. Direct micromorphological characterization of the three-dimensional physical gel network is not performed within this work. The mechanistic explanations associated with physical gel network structures presented below draw upon previously published literature, rather than direct microstructural experimental evidence obtained in the current investigation.

Under our fixed loading conditions, experimental observations reveal an apparent inverse trend between *β* and the characteristic macroscopic fatigue damage rate. Accordingly, *β* is treated as a lumped resistance-type kinetic index, and the physically consistent Arrhenius-type relationship is adopted to describe its temperature dependence:

(6) $$\ln\;(\beta )=\ln\;A^{\prime }+\frac{E_{a}}{RT}$$

where *A*′ is a lumped composite pre-exponential constant. It should be emphasized that this formulation and the resulting *E*_a_ are model-fitted apparent indicators only valid under the current fixed test conditions, rather than universal intrinsic material constants. Fitting ln*β* against (*RT*)^−1^ directly yields a slope equal to *E*_a_, and no post hoc absolute value operation is needed.

To determine the fatigue activation energy of the SBS asphalt mixture and the base asphalt mixture, *β* values measured under various temperatures are substituted into Equation (6). It is emphasized that *β* derived from Equation (1) is a lumped damage sensitivity exponent rather than a conventional chemical-reaction forward rate constant.

**Figure 3:**
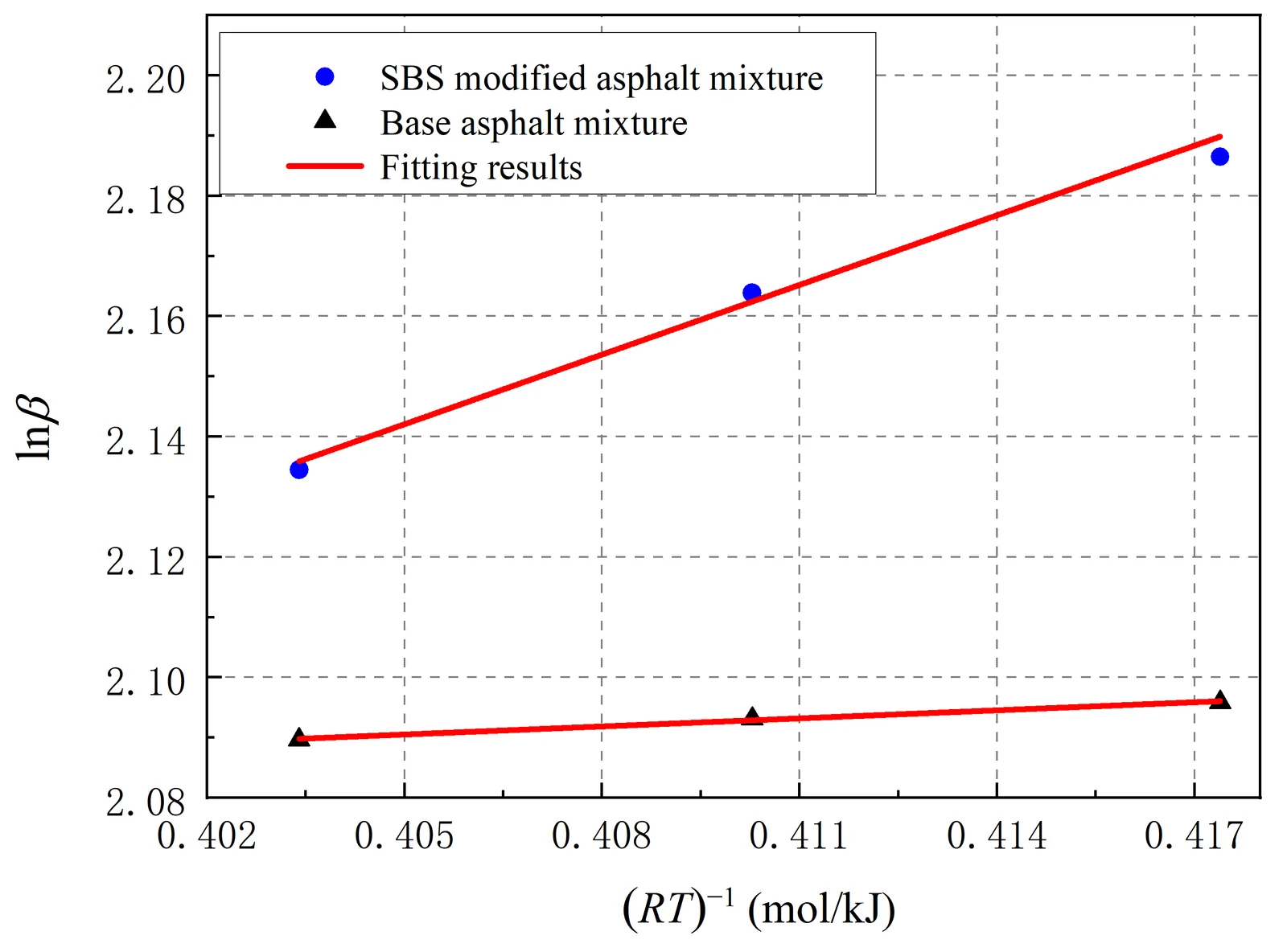
Matching results of fatigue damage activation energy for SBS and base asphalt mixtures.

As shown in Figure 3 and Table 2, the base asphalt mixture achieves a fatigue activation energy of 0.45 kJ·mol^−1^, whereas this parameter reaches 3.86 kJ·mol^−1^ for the SBS asphalt mixture. These results reveal that the SBS asphalt mixture demands a 3.41 kJ·mol^−1^ higher energy threshold to initiate fatigue damage compared with that of the base mixture. Meanwhile, its average fatigue life exceeds that of the base asphalt mixture by 5653 loading cycles. In other words, the SBS asphalt mixture shows higher resistance to fatigue damage initiation and longer service life performance. According to existing literature, such improvement may be associated with physical gel network structures originating from the SBS polymer.

The fatigue activation energy measured at the asphalt mixture scale deviates considerably from that reported at the binder scale in the literature [32], while the corresponding SBS and base asphalt binders yield activation energies of 59.92 kJ·mol^−1^ and 28.91 kJ·mol^−1^, respectively. This discrepancy originates from differences in material scale and loading configurations. Nevertheless, a consistent qualitative trend can be identified. SBS modification increases fatigue energy thresholds and prolongs fatigue life at both the binder and mixture scales, which corroborates the physical reasonableness of the present experimental findings.

## 3. Conclusions

A primary contribution of this study is extending the kinetics-based fatigue characterization framework from asphalt-binder scale to asphalt-mixture scale. Two kinetic indicators, including the lumped damage sensitivity exponent (*β*) and fatigue activation energy (*E*_a_), are proposed for the fatigue characterization of the SBS asphalt mixture. This framework is established under specific experimental conditions, namely, two mixture types (SBS and base asphalt mixtures), three temperatures (15, 20, and 25 °C), a fixed stress ratio of 0.3, and a constant frequency of 10 Hz via indirect tensile repeated loading tests. Within this scope, the main conclusions are as follows:

(1) Under cyclic loading, both fatigue damage and permanent strain in the SBS asphalt mixture exhibit a three-stage nonlinear evolution. A permanent strain model accounting for damage, developed within the framework of viscoelastic damage theory, captures both nonlinear mechanical responses with reasonable accuracy.
(2) A fatigue damage kinetic model of the SBS asphalt mixture, grounded in viscoelastic damage theory and chemical kinetics, incorporates two key kinetic parameters: the lumped damage sensitivity exponent and fatigue activation energy. These two parameters, respectively, characterize the overall rate and the energy barrier governing the variable-rate fatigue process under the tested conditions.
(3) Decreasing the test temperature leads to a systematic increase in the lumped damage sensitivity exponent *β*. Specifically, the values at 25 °C, 20 °C, and 15 °C are 8.45255, 8.70471, and 8.90380, respectively, corresponding to a total increase of 5.339% when the temperature is reduced from 25 °C to 15 °C.
(4) Under the tested conditions, the fitted kinetic parameter *E*_a_ for the SBS asphalt mixture (3.86 kJ·mol^−1^) is higher than that for the base asphalt mixture (0.45 kJ·mol^−1^). According to the published literature, such macroscopic difference may be interpreted with reference to the three-dimensional physical gel structures originating from the SBS polymer.

It should be noted that *α*, *ρ*, *γ*, *λ*, *κ*, and *β* are lumped composite coefficients consolidated from multiple physical quantities of the modified Burgers–Van der Poel viscoelastic framework. Complete formal term-by-term dimensional derivation and systematic dimensional consistency inspection for this set of lumped coefficients are complex and have not been fully performed within the current study. Systematic dimensional verification of the permanent strain model will be addressed in future research.

It should be noted that the *E*_a_ presented herein is a model-fitted kinetic indicator obtained under limited test conditions. Direct experimental quantification of intrinsic damage-initiation energy within asphalt mixtures remains experimentally challenging. Further investigations are required to enrich and refine this theoretical framework for kinetics-based fatigue characterization.

It should be noted that this study mainly aims to reproduce measured permanent strain curves and investigate parameter variations across temperatures and mixture types, instead of assessing the model’s out-of-sample prediction for unseen loading conditions. Further cross-validation with independent datasets and systematic parameter uncertainty and sensitivity analyses will be performed in future work. Additional temperature points (e.g., 10 °C and 30 °C) are recommended for future work to further validate the estimated *E*_a_. It should be noted that all fatigue tests in this work were carried out at a fixed stress ratio of 0.3 and a fixed loading frequency. Accordingly, the fatigue kinetic parameters *β* and *E*_a_ obtained in this study are valid only for this specific loading condition, rather than intrinsic universal material constants independent of stress level and loading frequency. These parameters may change if the stress ratio or loading frequency varies. Further multi-stress-ratio and multi-frequency laboratory tests are required in future investigations to reveal the loading-dependent characteristics of *β* and *E*_a_.

## 4. Materials and Methods

### 4.1. Test Materials

The styrene–butadiene–styrene (SBS) modifier is a triblock copolymer. Its polystyrene end blocks physically crosslink to form aggregated domains within the base asphalt, whereas the polybutadiene mid-block constitutes the continuous phase. This arrangement gives rise to a thermally reversible physical gel network that substantially enhances the toughness of the asphalt material. The commercial SBS asphalt binder used in this study contains 4.5% SBS modifier by weight of the binder, with the base asphalt serving as the reference. The fundamental properties of both binders are summarized in Table 3. Granite was selected as the coarse aggregate, and the gradation adopted was Stone Mastic Asphalt (SMA), which is a mixture type widely employed in pavement engineering. The relevant mixture design parameters are presented in Table 4.

### 4.2. Specimen Preparation of SBS Asphalt Mixture

**Figure 4:**
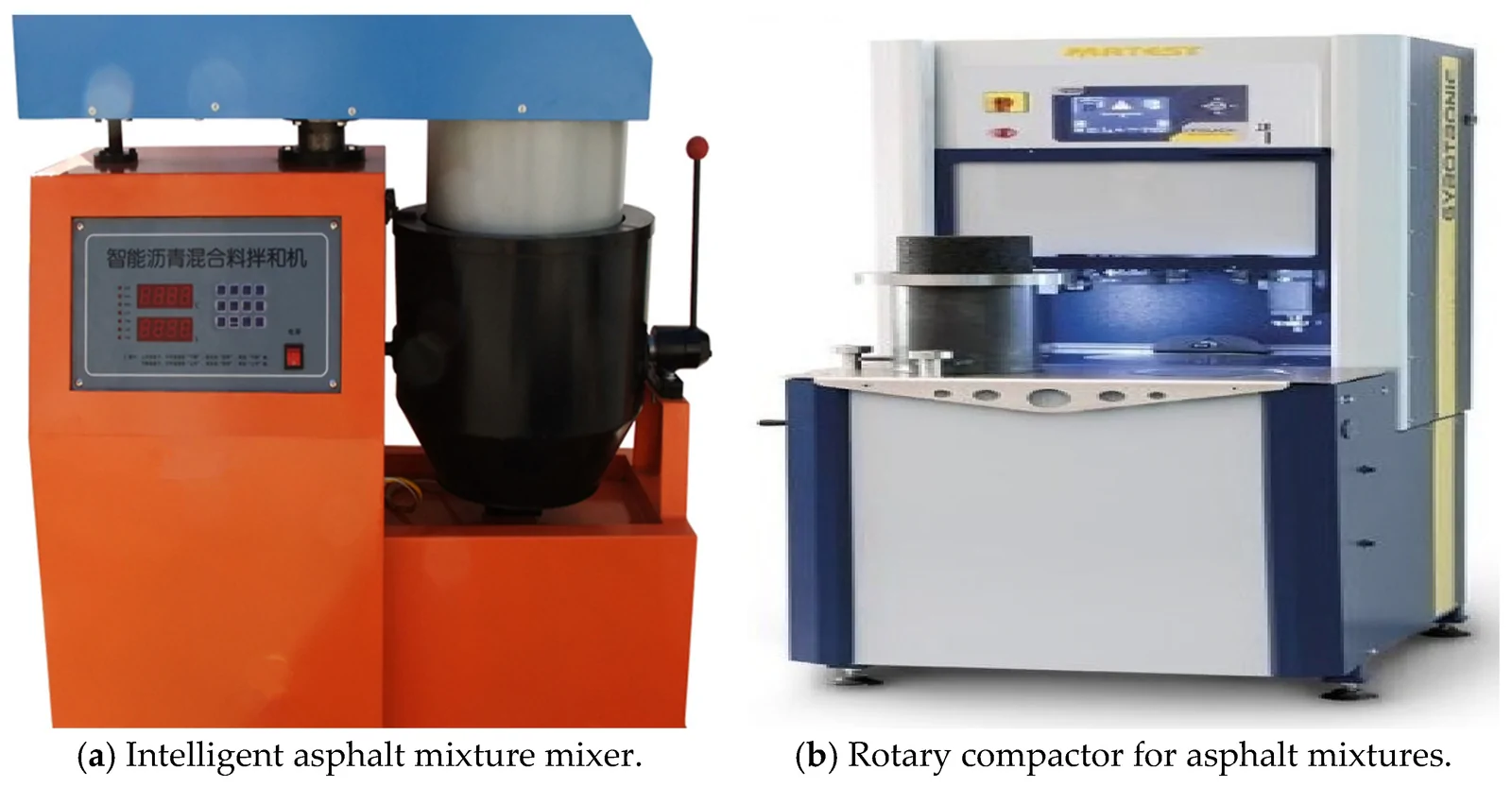

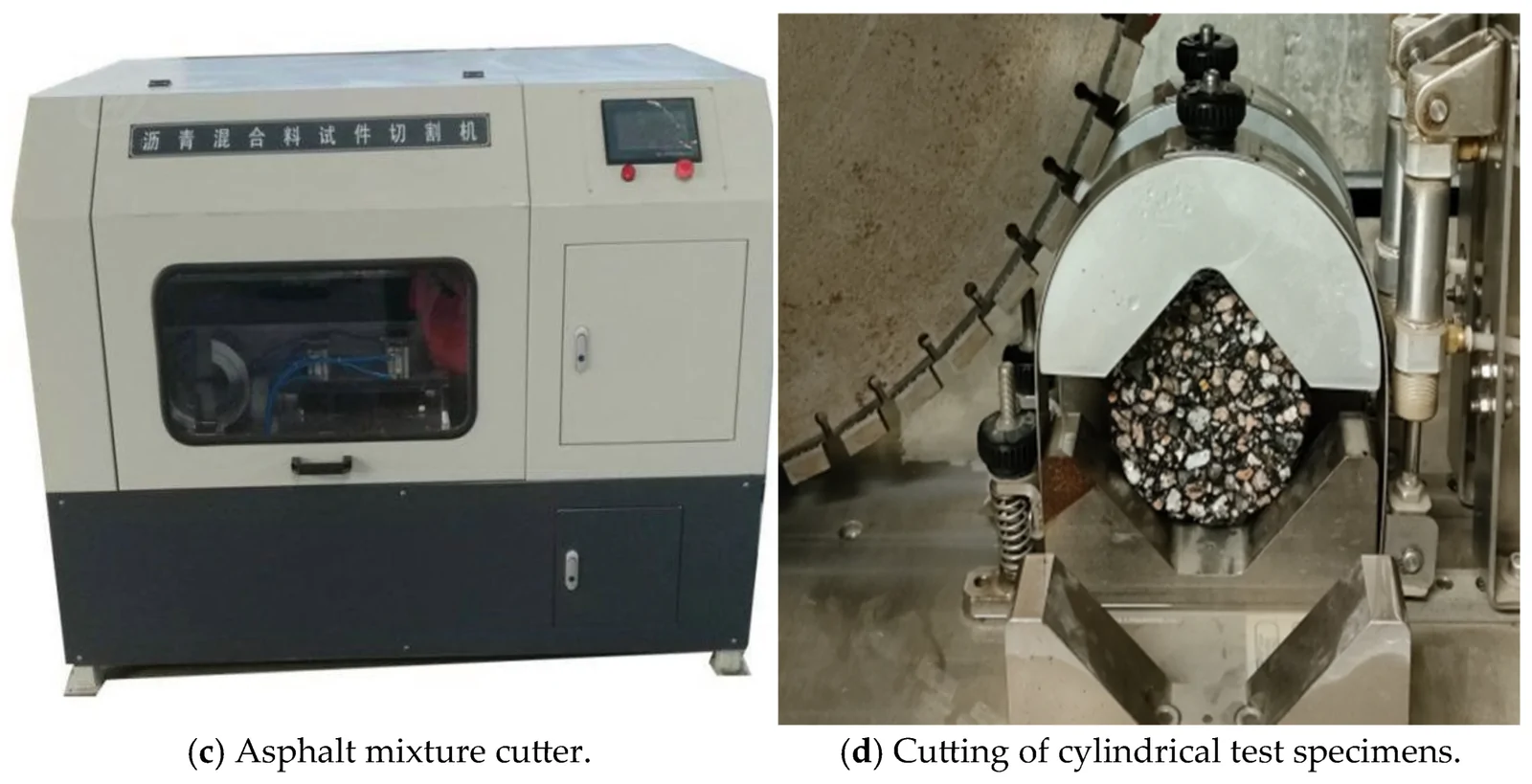
Molding and cutting of asphalt mixture specimens.

Based on the designed asphalt content of 6.0% by weight of the mixture, the aggregates and asphalt binder were accurately weighed. The aggregates were preheated to 175 °C and then transferred into an intelligent asphalt mixture mixer, shown in Figure 4a, where they were mechanically blended at 175 ± 5 °C for approximately 90 s to ensure uniform coating of the aggregates by the asphalt binder. The resulting mixture was then placed into a cylindrical metal mold with a diameter of 100 mm and compacted using a Superpave gyratory compactor shown in Figure 4b under a vertical pressure of 600 kPa, an internal angle of 1.25°, and a gyration speed of 30 rpm. The number of gyrations was adjusted to achieve a final specimen height of 150 mm. Throughout the compaction process, the air void content was monitored in real time using the volumetric method and strictly controlled within the range of 4% ± 0.5%.

The compacted specimens were cooled with an electric fan for 30 min prior to demolding. Approximately 15 mm was precisely sawn off from each end of the 150 mm high compacted cylinder using a dedicated double-face saw to remove uneven compaction boundary zones and ensure flat cut surfaces perpendicular to the longitudinal axis, as shown in Figure 4c,d. After end trimming, the remaining homogeneous intermediate section of the sample was further sawn into segments. This multi-step trimming and segmenting procedure yielded cylindrical specimens with a diameter of 100 mm and a height of 40 mm, which were subsequently used for mechanical performance testing. Throughout the entire fabrication process, careful attention was paid to temperature control, compaction effort, and dimensional accuracy to ensure internal uniformity and density of the final specimens.

### 4.3. Indirect Tensile Monotonic and Repeated Loading Tests of SBS Asphalt Mixture

**Figure 5:**
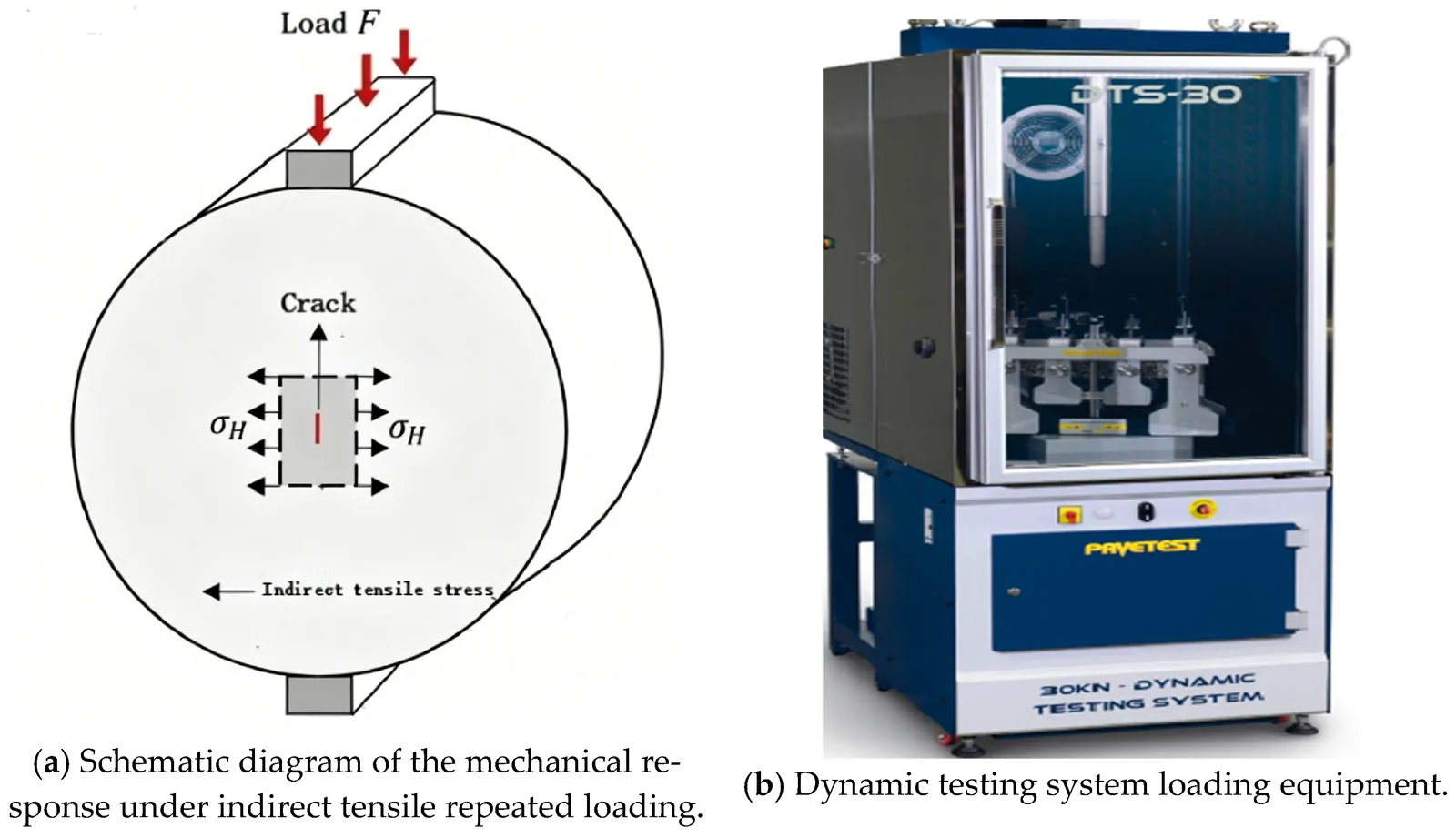
Schematic diagram of an indirect tensile fatigue test.

The indirect tensile repeated loading test enables precise measurement of the strain response of asphalt mixtures using displacement transducers, as shown in Figure 5a. Furthermore, this testing approach possesses prominent merits, such as simple specimen preparation, high test reproducibility, and convenient field application, making it widely utilized within pavement engineering. Therefore, the indirect tensile repeated loading test was selected to determine the variable-rate fatigue process of the SBS asphalt mixture. The dynamic test system illustrated in Figure 5b served as the loading equipment for both the indirect tensile monotonic tests and the indirect tensile repeated loading tests. The loading frequency was set to 10 Hz, corresponding to a vehicle speed of 60–65 km/h. Experiments were carried out at intermediate temperatures of 15 °C, 20 °C, and 25 °C, representing typical service conditions susceptible to fatigue-related cracking. Five replicate tests were performed for each testing condition.

#### 4.3.1. Indirect Tensile Monotonic Loading Test of SBS Asphalt Mixture

To determine the appropriate load amplitude for the indirect tensile repeated loading test on the SBS asphalt mixture, indirect tensile monotonic loading tests were first conducted on the specimens. These tests were performed under a displacement-controlled mode at a constant loading rate of 50 mm/min.

**Figure 6:**
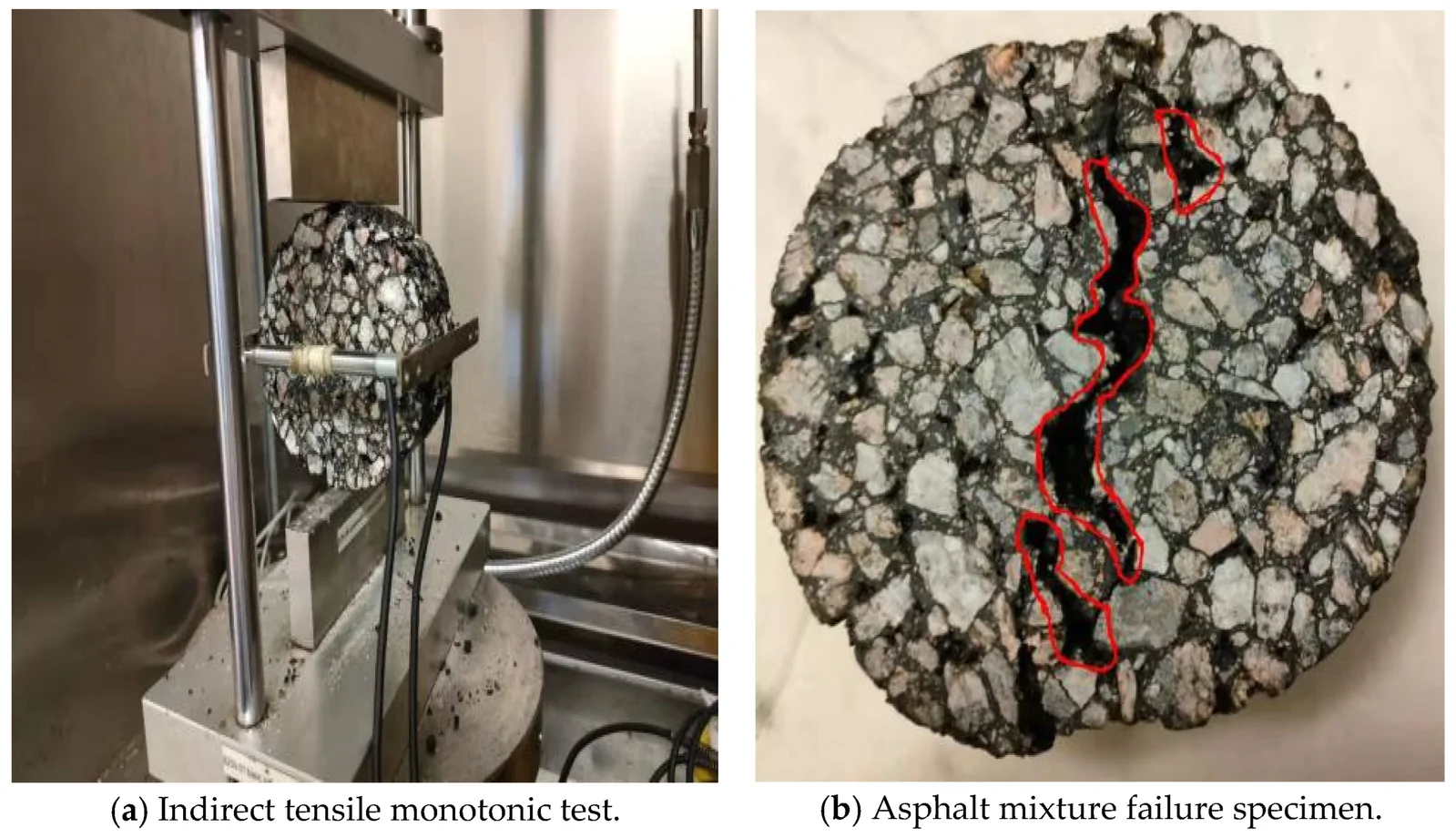
Indirect tensile monotonic test and failure specimen.

First, two perforated steel plates were vertically attached to opposite sides of the specimen surface using an epoxy structural adhesive as the bonding agent. Two displacement transducers were then inserted into the perforations of the steel plates to capture the transverse tensile strain signals generated during loading in the dynamic testing system, as shown in Figure 6a.

Afterward, the specimen was centered on the support fixture and transferred into the thermostatic cabinet for 2 h of thermal pre-conditioning under the target test temperature. Afterward, the dynamic test setup was initiated to exert axial compressive loading on the specimen at 50 mm/min, continuing until fatigue fracture took place, as shown in Figure 6b. The peak failure load (*F*_max_) was documented. The approximate tensile stress of the specimen was computed via the formula reported in reference [26]. The stress value corresponding to (*F*_max_) was regarded as specimen strength (*σ*_max_). The obtained strength data are compiled in Table 5.

Five replicate specimens were prepared and tested for each testing condition. The tabulated fatigue life results are arithmetic mean values of the parallel tests. The standard deviation (SD) and the coefficient of variation were adopted to quantify the experimental data scatter. Given that only five replicates were available for each group, formal inferential statistical significance analysis was not performed. Comparisons across temperatures and mixture types were therefore made by comprehensive evaluation of mean values alongside dispersion indicators, including the SD and the coefficient of variation.

(7) $$\sigma =\frac{2F}{\pi \;t\;\Omega }$$

where *Ω* is the specimen diameter (mm), *t* is the specimen height (mm), and *F* is the applied force (*N*).

#### 4.3.2. Indirect Tensile Repeated Loading Test of SBS Asphalt Mixture

Indirect tensile repeated loading tests were conducted to capture the variable-rate fatigue process of the SBS asphalt mixture. The repeated loading tests were performed in stress-controlled mode with a haversine waveform at a stress ratio of 0.3, corresponding to a cyclic load amplitude of 0.3*σ*_max_. The dynamic test setup imposed repeated loads on the specimen until fatigue failure.

**Figure 7:**
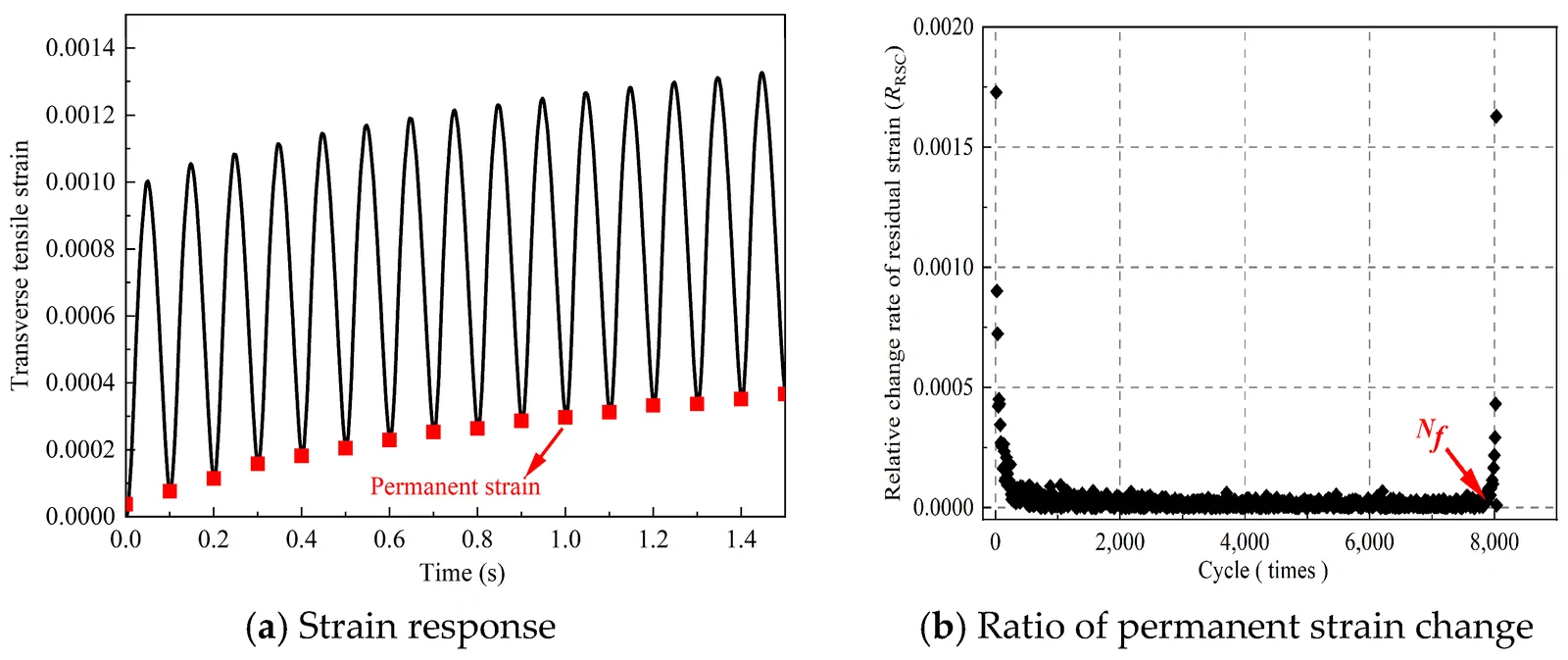
Strain response and ratio of permanent strain change.

When subjected to repeated loading, the total strain of the SBS asphalt mixture consists of three parts, namely, elastic, viscoelastic, and viscoplastic strain. Once unloading commences, elastic strain can be completely restored, whereas viscoplastic strain cannot be recovered, and viscoelastic strain exhibits partial reversibility only. The transverse tensile strain measured at the valley of every loading cycle is regarded as the permanent strain (*PS*) for the SBS asphalt mixture, and its development curve is illustrated in Figure 7a.

**Figure 8:**
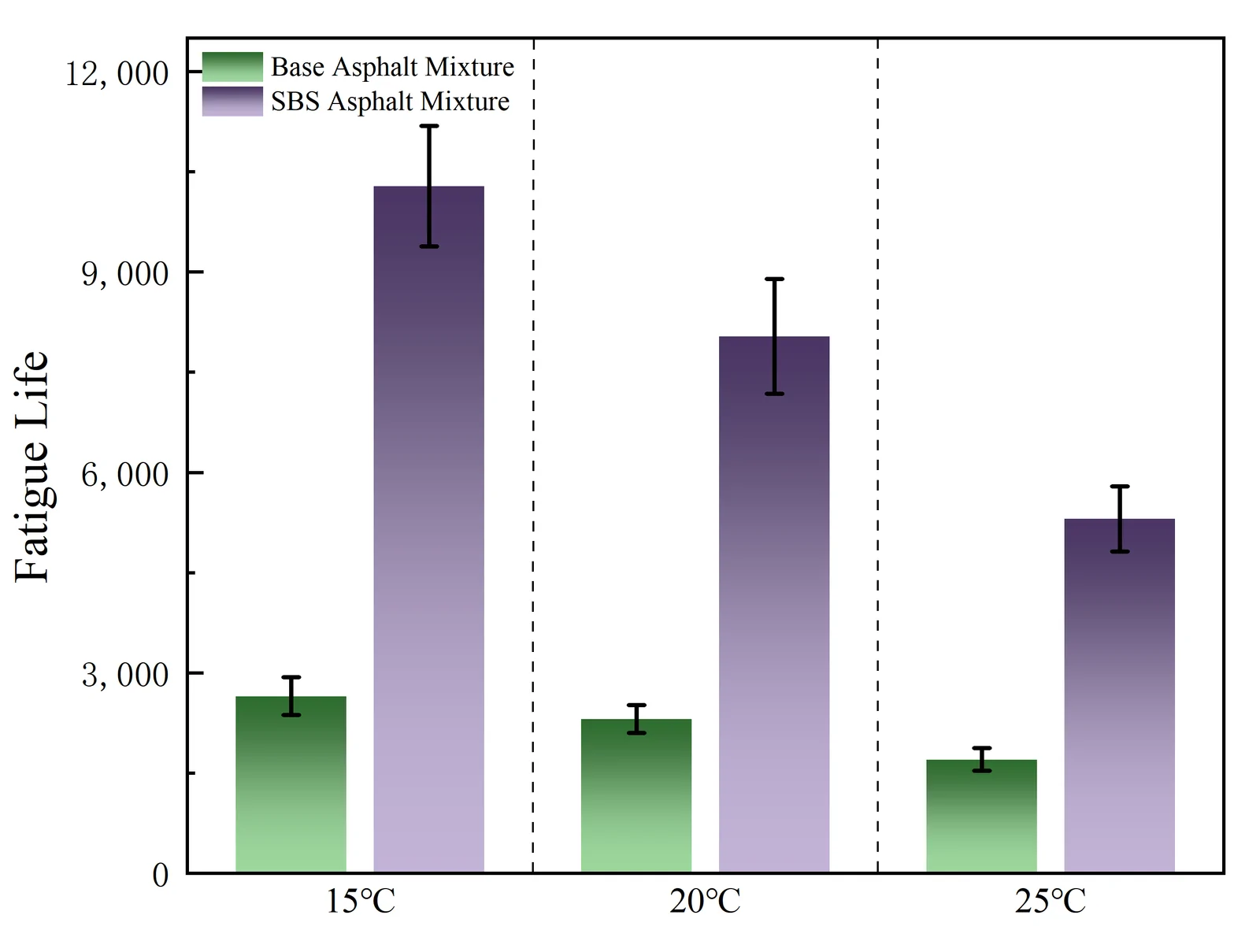
Fatigue life results.

The ratio of permanent strain change (*RPSC*) of the SBS asphalt mixture was calculated using Equation (8), and the resulting *RPSC* curves are presented in Figure 8. *RPSC* serves as an indicator reflecting the anti-damage capacity of asphalt mixtures under cyclic loading. The transition point between the secondary and tertiary stages of the *RPSC* curve marks the critical state at which the specimen substantially loses cracking resistance capability owing to continuous accumulation of internal fatigue damage. The number of loading cycles corresponding to this transition point is defined as the fatigue life (*N_f_*) [17,26], as shown in Figure 7b. This experimental failure criterion based on *RPSC* evolution establishes a practical, measurable threshold linked to the physical meaning of fatigue damage, in which progressive growth of the internal defect area (described by *D*) drives the evolution of permanent strain and finally triggers the stage transition observed on the *RPSC* curve. The fatigue life results obtained under various testing conditions are summarized in Table 5 and Figure 8.

(8) $$RPSC=\frac{PS_{N+1}-PS_{N}}{PS_{N}}$$

where *PS_N_* is the permanent strain at the *N*-th loading cycle.

It should be noted that fatigue tests adopted an identical stress ratio (30% of tensile strength) rather than identical absolute stress amplitude. Due to large differences in tensile strength between SBS-modified and base asphalt mixtures, applied absolute stress differs for each group. Raw fatigue life values cannot be unambiguously compared directly because differences may arise from both material fatigue properties and loading-stress variation. Accordingly, core analysis relies on normalized kinetic parameters instead of fatigue life.

### 4.4. Development of Permanent Strain Response Model of SBS Asphalt Mixture Based on Viscoelastic Damage Theory

**Figure 9:**
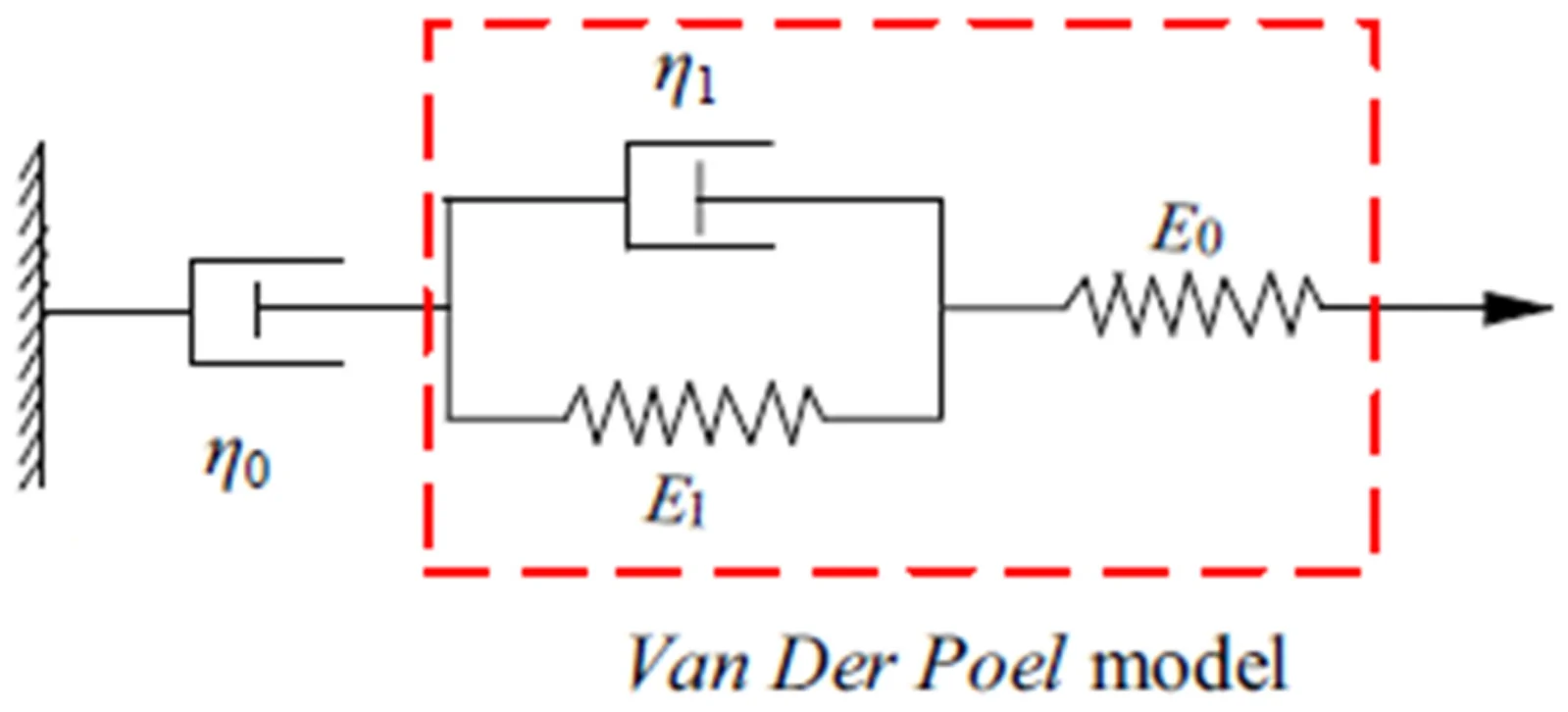
Burgers model.

According to viscoelastic theory, the permanent strain of the SBS asphalt mixture can be described by the Burgers model, as shown in Figure 9.

Relevant literature [26,27] indicates that by modifying the series visco-pot model with a binary function as shown in Equation (9), it is possible to describe the variation patterns of the permanent strain in SBS asphalt mixture across three stages in a relatively comprehensive and accurate manner. This yields a model for permanent strain (*ɛ*_p,*N*_) that does not account for damage, as shown in Equation (10)

(9) $$\eta_{0}(t)=\frac{\eta_{0}}{at^{2}+bt+1}$$

where *a* and *b* are constants, and *η*_0_ is the initial viscosity coefficient.

(10) $$\varepsilon_{p,N}=\alpha N^{3}+\rho N^{2}+\gamma N+\lambda (1-e^{-\kappa TN})$$

The above model is capable of representing the permanent strain response of the SBS asphalt mixture under intact conditions. Nevertheless, this model fails to consider how fatigue damage influences permanent strain accumulation within such SBS mixture. Accordingly, it is necessary to establish a permanent strain model that incorporates damage effects for SBS asphalt mixture [28,29].

During the fatigue damage evolution, the effective stress can be expressed as [30,31]:

(11) $$\tilde{\sigma }=\frac{\sigma }{1-D}$$

where here σ denotes Cauchy stress, and $\tilde{\sigma }$ denotes effective stress.

The baseline damage-free permanent strain model shown in Equation (10) is adopted from published literature [26,27,28], which describes the viscoplastic response of asphalt mixtures under intact conditions without internal fatigue deterioration.

During fatigue damage evolution, progressive internal defects reduce the effective load-bearing area of the material matrix according to the effective stress assumption, and the actual stress acting on the material matrix is modified by the damage variable $D$. Substituting the effective stress formulation into the damage-free permanent strain model yields the coupled permanent strain model accounting for fatigue damage effect, as shown in Equation (12).

(12) $$\varepsilon_{p,N}=\frac{\varepsilon_{p,N}}{1-D}=\frac{\alpha N^{3}+\rho N^{2}+\gamma N+\lambda (1-e^{-\kappa TN})}{{\left(1-\frac{N}{N_{f}}\right)}^{\frac{1}{1+2\beta }}}$$

Definitions of composite coefficients in the permanent strain model are shown in Table 6. Taking one set of fitted data of the SBS asphalt mixture at 15 °C as a concrete example to illustrate what each parameter represents. The permanent strain model shown in Equation (10) represents the original damage-independent formulation. Structurally, it contains no damage-related terms and is only valid for asphalt mixture materials under undamaged conditions. As a result, it cannot reflect the progressive material deterioration induced by accumulating fatigue damage and cannot account for the accelerated tertiary stage growth of permanent strain under cyclic loading. By contrast, the fatigue damage model in Equation (2) focuses purely on damage evolution and lacks the capability to calculate permanent strain magnitudes.

Compared with these two independent formulations, Equation (12), proposed in this paper incorporates an additional damage-coupled term derived from the effective stress assumption. By embedding the damage variable into the permanent strain modelling framework, the newly developed model can simultaneously describe fatigue damage evolution and capture the full range permanent strain development of the SBS asphalt mixture throughout cyclic loading. From the physical modelling perspective, the introduction of this damage-coupled term improves predictive performance for the three stage nonlinear permanent strain behavior under fatigue loading.

It should be highlighted that *α*, *ρ*, *γ*, *λ*, *κ,* and *β* are lumped composite coefficients inherited from the modified Burgers–Van der Poel viscoelastic damage framework rather than arbitrary empirical tuning parameters. Each coefficient corresponds to an independent physical mechanism governing permanent strain and fatigue damage evolution. Despite obvious magnitude differences among parameters, removing any mechanical term will impair the model’s capability to capture the three-stage permanent strain characteristics of asphalt mixtures. The tiny-magnitude *γ* term is reserved for theoretical completeness, even though its contribution is negligible within the tested loading-cycle range. Due to an insufficient number of complete replicated permanent strain curves for each test condition, formal quantitative confidence intervals for fitted parameters are not provided in this study. Systematic identifiability and a parameter-uncertainty analysis will be performed with richer replicate datasets in future investigations. In addition, complete term- by-term dimensional derivation for these lumped composite coefficients remains a subject for follow-up research.

## Figures and Tables

**Table 1:** Matching results for permanent strain model accounting for damage.

Asphalt Mixture	Temperature (°C)	*α*	*ρ*	*γ*	*λ*	*κ*	*β*	*R* ^2^	RMSE	MAE
SBS Asphalt Mixture	15	4.32 × 10^−6^	−5.83 × 10^−10^	3.50 × 10^−14^	0.00071	0.00441	8.90380	0.995	0.00065	0.00051
	20	5.18 × 10^−6^	−7.23 × 10−10	5.83 × 10^−14^	0.00100	0.00686	8.70471	0.987	0.00056	0.00043
	25	8.90 × 10^−6^	−1.88 × 10^−9^	2.29 × 10^−13^	0.00112	0.01040	8.45255	0.971	0.00060	0.00046
Base Asphalt Mixture	15	2.19 × 10^−5^	−1.14 × 10^−8^	2.65 × 10^−12^	0.00101	0.01707	8.13260	0.998	0.00052	0.00041
	20	2.39 × 10^−5^	−1.16 × 10^−8^	3.25 × 10^−12^	0.00128	0.02336	8.11030	0.997	0.00045	0.00036
	25	3.38 × 10^−5^	−2.21 × 10^−8^	8.41 × 10^−12^	0.00133	0.03161	8.08222	0.997	0.00038	0.00031

**Table 2:** Results for lumped damage sensitivity exponent and fatigue activation energy.

Asphalt Mixture	Temperature (°C)	(*RT*)^−1^ (mol/kJ)	Fatigue Life (Cycles)	*β*	Residuals *e_i_*	*E*_a_ (kJ·mol^−1^)	*RMSE*	*R* ^2^
SBS Asphalt Mixture	15	0.41739	10,285	8.90380	0.00254	3.86	0.00530	0.972
	20	0.41028	8035	8.70471	0.00737			
	25	0.40340	5306	8.45255	0.00483			
Base Asphalt Mixture	15	0.41739	2653	8.13260	0.00171	0.45	0.00193	0.991
	20	0.41028	2310	8.11030	0.00217			
	25	0.40340	1705	8.08222	0.00187			

**Table 3:** Fundamental properties of asphalt binders.

Indicators	SBS Asphalt	Base Asphalt
Viscosity (135 °C) (Pa·s)	1.54	1.84
Flash point (°C)	308	315
Solubility (%)	96.83	99.97
Softening point (°C)	54.9	44.2
Penetration (25 °C) (0.1 mm)	57	69

**Table 4:** Aggregate gradation.

Ingredients	Relative Density (g/cm^3^)	Dimensions (mm)	Percentage (%)
Coarse-grade material	2.642	14–10	3.5
	2.663	10–5	59.5
	2.709	5–2.36	9.0
Fine-grade material	2.649	2.36–0.075	16.5
Filler	2.661	<0.075	9.5
Lime	2.587		2.0

**Table 5:** Test specimen strength and fatigue life results.

Asphalt Mixture	Temperature (°C)	Specimen Strength (MPa)	Fatigue Life (Cycles) Mean ± SD	Coefficient of Variation (%)
Base Asphalt Mixture	15	0.361	2653 ± 283	10.672
	20	0.241	2310 ± 207	8.971
	25	0.184	1705 ± 168	9.891
SBS Asphalt Mixture	15	0.598	10,285 ± 903	8.781
	20	0.355	8035 ± 858	10.679
	25	0.268	5306 ± 492	9.279

**Table 6:** Definitions of the fitted composite coefficients in the permanent strain model.

Symbol	Physical Significance
*α*	Composite coefficient transformed from modified dash-pot parameters and loading conditions, characterizing the first-order contribution of viscoplastic flow to permanent strain accumulation with increasing loading cycles. For the SBS asphalt mixture at 15 °C, *α* = 4.32 × 10^−6^ reflects the baseline magnitude of viscoplastic deformation growth.
*ρ*	Composite coefficient consolidated from viscoelastic parameters and loading conditions, characterizing the second-order coupling effect between internal material deterioration and viscoplastic flow as loading cycles accumulate. For SBS asphalt mixture at 15 °C, ***ρ*** = −5.83 × 10^−10^ indicates a weak corrective effect of damage on viscoplastic strain.
*γ*	Higher-order composite coefficient originating from modified dash-pot constitutive terms, describing the third-order nonlinear coupling between progressive material deterioration and viscoplastic-strain evolution. For SBS asphalt mixture at 15 °C, ***γ*** = 3.50 × 10^−14^ is extremely small, meaning this third-order term provides a negligible contribution within the tested loading cycle range.
*λ*	Composite coefficient converted from Van der Poel creep-compliance parameters, governing the evolution amplitude of recoverable residual viscoelastic strain under cyclic loading. For SBS asphalt mixture at 15 °C, λ = 0.00071 quantifies the magnitude of the recoverable viscoelastic deformation.
*κ*	Composite coefficient derived from the Van der Poel model, controlling the decay rate of recoverable viscoelastic component as damage accumulates with loading cycles. For SBS asphalt mixture at 15 °C, *κ* = 0.00441 represents how fast residual viscoelastic strain dissipates.
*β*	Lumped damage sensitivity exponent obtained from the fitting of dissipated-energy-based damage-evolution relation, quantifying the overall evolution rate of fatigue damage over loading cycles. For SBS asphalt mixture at 15 °C, *β* = 0.00441 characterizes the overall rate of fatigue damage accumulation.

## Data Availability

Data are contained within the article. Some or all of the data, models, or code that support the findings of this study are available from the corresponding author upon reasonable request.
